# Individual differences in sensory processing sensitivity amplify effects of post-learning activity for better and for worse

**DOI:** 10.1038/s41598-023-31192-9

**Published:** 2023-03-17

**Authors:** Robert Marhenke, Bianca Acevedo, Pierre Sachse, Markus Martini

**Affiliations:** 1 Department of Psychology, University of Innsbruck, 6020 Innsbruck, Austria; 2 Department of Psychological and Brain Sciences, University of California, Santa Barbara, Santa Barbara, CA 93106-5060 USA

**Keywords:** Psychology, Risk factors

## Abstract

Sensory processing sensitivity (SPS) is a biologically-based trait associated with greater reactivity to both positive and negative environments. Recent studies suggest that the activity following learning can support or hinder memory retention. Here, we employed a within-subject experiment to examine whether and how individual differences in SPS contribute to differences in memory retention. Sixty-four participants encoded and immediately recalled two word lists: one followed by 8-min of eyes-closed, wakeful resting; and the other by a distraction task. After 7 days, participants completed a surprise free recall test for both word lists. If participants wakefully rested after encoding, memory retention increased as a function of higher SPS. However, in the distraction condition, a negative curvilinear relationship indicated that memory retention was especially hindered for highly sensitive individuals. These results suggest that individual differences in SPS are an important factor to consider when examining the effects of environmental conditions on learning and memory.

## Introduction

Our ability to encode, store and retrieve information varies vastly depending on environmental and individual factors^1^. While contextual/environmental influences on intraindividual differences in learning and memory are the subject of intense study, interindividual differences in temperament and their interactions with environmental factors have often been overlooked in modern learning and memory research, particularly with respect to long-term memory.

### Wakeful resting

The period immediately after learning new information is critical for successful long-term memory retention^2,3^. A nascent body of research indicates that eyes-closed, wakeful resting immediately following the encoding of new information may support memory retention compared to an equivalent period of active wake such as engaging in a new task^4^. Such an *offline*, eyes-closed, state of rest may alleviate demand on hippocampal (and other) resources, providing an opportunity to consolidate newly formed memory traces^5^. In contrast, any form of *online*-processing (i. e., mentally effortful task) following encoding of information has detrimental effects on memory retention^3^. Even though many studies have found beneficial effects of wakeful resting on memory retention relative to other attention-intensive tasks, for a variety of different learning materials^6^; several recent studies failed to replicate these effects or only replicated results under very specific conditions^7–13^. Moreover, effect sizes vary considerably within, as well as between, samples ranging from very large differences to null effects (see Humiston et al.^13^ for meta-analyses). In addition to justified criticism regarding studies sometimes being underpowered to detect subtle effects of rest on memory^13^, we herein propose that conflicting results could also be attributable to inherent individual differences between different subjects of studied samples. Perhaps resting after encoding new information is only beneficial or especially beneficial for some, but not all individuals.

Indeed, neural activity during wakeful resting states vary across individuals, and appears to be predictive of individual differences in memory ability^14^. Similarly, neural activity during rest is also associated with individual differences in a biologically-based trait often referred to as sensory processing sensitivity (SPS) or environmental sensitivity (for review see Acevedo et al.^15^).

### Sensory processing sensitivity

SPS is proposed to be a common, evolutionarily conserved trait associated with greater depth of processing and more reflective information processing, lower sensory thresholds to stimulation, ease of overstimulation and higher emotional and physiological reactivity^16,17^. This concept is based on a large foundation of biological evidence, demonstrating that within many species, from fish to humans, two conversing behavioral patterns or ‘strategies’ consistently evolved for responding to novel environmental information^18,19^. While some individuals are more inclined to inhibit behavior to allow for deeper processing of new information (‘geared to inspect’); others tend to act more impulsively, thus are faster, but less reflective in their responses (‘geared to respond’)^20^. These strategies map well onto SPS, as those with higher levels of the trait express the cautious and reflective behavioral strategy, which can be more easily described in terms of either responding more to the environment versus responding less^21^. Although, it is still a matter of debate whether these two strategies may in fact be continuous, some work on infant reactivity and population studies with adults suggest that roughly 20 to 30% of individuals in many species tend to be high in SPS or express a more responsive, cautious behavioral strategy^17,22,23^. Contrary to previous theories assuming that higher sensitivity would only be a vulnerability factor to adverse environmental conditions^24^; other theories, like the *Biological Sensitivity to Context Theory*^19^ and the *Environmental Sensitivity* framework^25^ posit that more sensitive individuals also experience stronger effects and responsivity to positive environmental factors, both for better and for worse. Thus, higher SPS individuals may experience greater benefits in response to positive conditions, but also more adverse outcomes under negative conditions^26^.

Consistent with theory and research proposing that depth-of-processing is a central feature of SPS^16,17^, Acevedo et al.^27^ found enhanced patterns of resting state brain connectivity related to depth of processing but also patterns associated with memory-consolidation, when participants rested for five minutes after engaging in an empathy task. The results showed that in addition to the expected, enhanced resting-state brain connectivity within the ventral attention, dorsal attention and limbic (emotional processing) networks; notable enhanced connectivity was also shown in the default mode network between the hippocampus and the precuneus as a function of greater SPS. The hippocampus is essential in short-term memory retention and long-term memory consolidation through the gradual integration of memory traces into more stable neocortical networks during resting states^28^. The precuneus, as one of those neocortical areas, is often seen as a central area for the storage of episodic long term memory^29^. Heightened connectivity between these structures could be interpreted as a physiological marker for the transfer of information from short- to long-term memory storage^30,31^. Thus, Acevedo et al.’s^27^ results could be a first neuroscientific indicator that higher SPS individuals indeed show deeper and more reflective information processing, and that this reflectivity not only occurs in often suspected attention and emotion-related networks, but also might be part of a more elaborative memory processing strategy. This is well in line with studies showing that more introverted individuals tend to spend more time reflecting on punishing feedback to avoid future mistakes^32^. However, since the default mode network is most active during wakeful resting states and consistently decreases its activity during active online-processing of external factors (e.g. goal directed tasks)^33^, greater depth of processing and memory related resting-state connectivity within the default mode network might also depend on individuals’ activities.

Deeper and more elaborative processing of stimuli leads to longer lasting memory traces^34^, which can then be reactivated and further consolidated by hippocampal replay or hippocampal–cortical interactions during offline periods^35,36^. This benefit, however, comes at the cost of greater mental effort. With increasing effort invested in stimulus processing, spare processing capacity could be reduced^37^. Thus, as higher SPS individuals tend to engage in deeper, more reflective/elaborative information processing, they also have to exert greater effort to do so. When processing demands intensify, there is an increasing discrepancy between demand and supply of effort, and higher (vs. lower) SPS individuals might invest more effort in the current activity. Thus, they might have less spare processing capacity for subconscious memory retention. This is in accordance with frequent reports of high SPS individuals being easily overwhelmed by highly stimulating environments and preferring to focus on one task at a time^16^. Thus, if higher SPS individuals have to exert greater effort in order to perform a learning task, they ought to have less spare capacity available to them than lower SPS individuals to cope with additional attentional demands, imposed by environmental stimulation^38^. Higher SPS individuals should thus be more negatively affected by distracting stimuli. Indeed, at least one study has shown that individuals with higher SPS performed better at a visual detection task, but that they also reported higher stress levels after engaging in the task^39^.

### The present study

Consequentially, we assume that wakeful, “offline” resting periods with one’s eyes closed in comparison to “online” periods of active wake might affect individuals’ memory retention differently, as a function of their SPS. While higher SPS individuals might benefit more from wakeful resting after learning, by allowing for deeper offline-cognitive processing, they might also be more negatively affected by distracting stimulation during active wake.

To this end, the first goal of the present study was to test whether post-encoding wakeful rest benefits memory retention for a word list to a greater extent than engaging in an attention-demanding distraction task, as was the case in some but not all previous studies^13^. However, as the first study investigating SPS in the context of memory and learning, the second and primary aim of this study was to investigate whether and how individual differences in SPS moderate memory retention. We expected that SPS would moderate memory retention differently, depending on the activity following new learning. Specifically, we expected that higher (vs lower) SPS individuals would show better memory retention for a word-list, if they were allowed to wakefully rest after encoding, but worse memory retention, if they would have to perform a post-encoding attention-demanding distraction task.

## Method

### Participants

This research was approved by the Board for Ethical Questions in Science of the University of Innsbruck and all procedures adhered to the ethical principles for research with humans. All data collection and reporting associated with this manuscript were carried out in compliance with internationally-accepted standards for research practice and reporting according to the Committee on Publication Ethics’ (COPE) guidelines. Sixty-four students (37 females, 27 males; mean age = 23.73 years, age range = 19–52 years) participated in this experiment in exchange for partial course credit. Six participants did not return for Session 2. They were replaced in the sampling plan and excluded from analyses. All participants were briefed and provided informed consent in writing prior to their participation.

### Materials and procedure

Similar to previous experiments on wakeful resting^4,11^ each participant completed two testing sessions, Session 1 and Session 2, which were separated by 7 days.

**Figure 1:**
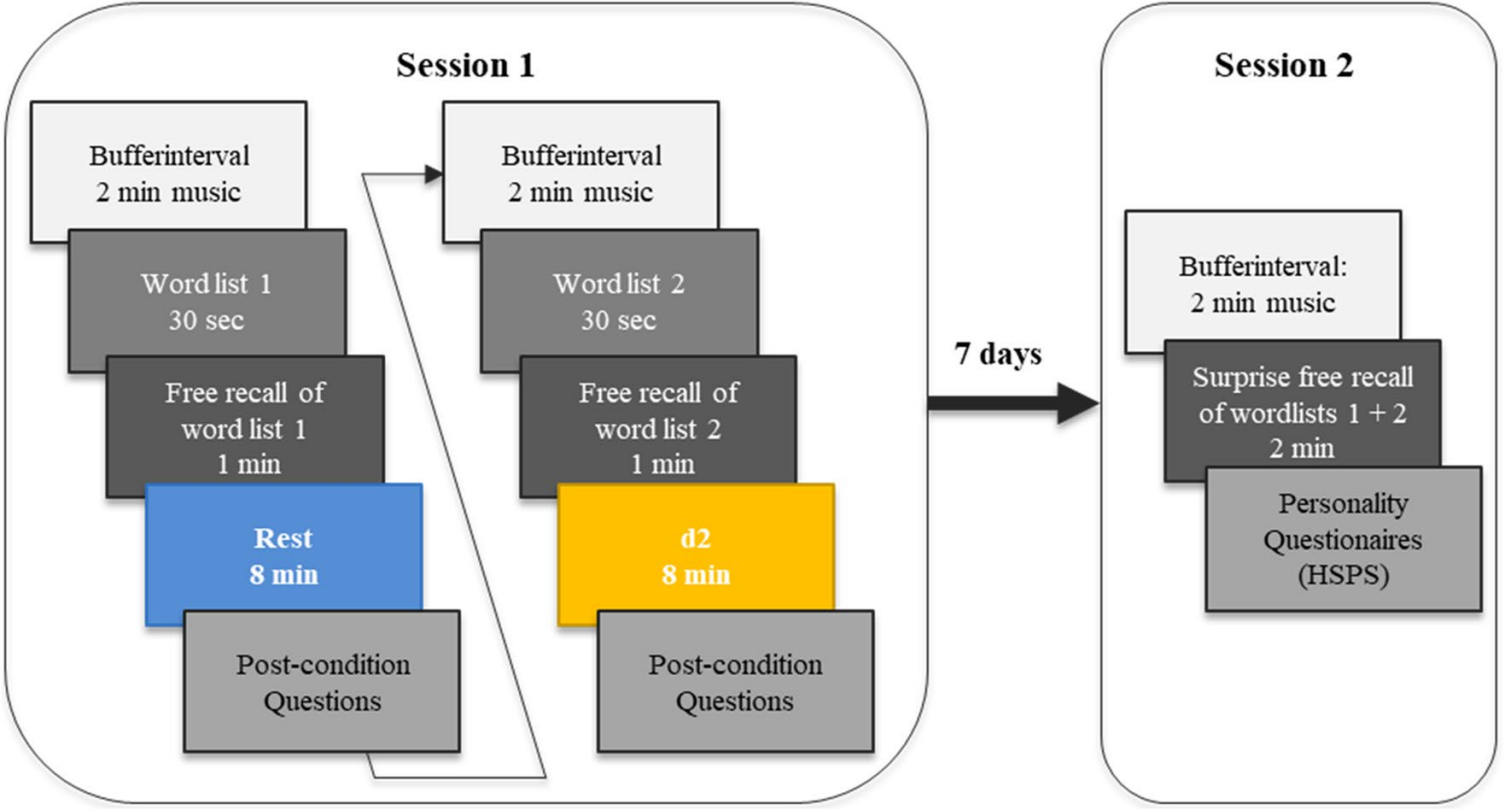
Experimental procedure. The experiment consisted of two experimental sessions (Session 1 and 2) separated by 7 days. In Session 1, participants completed two word-learning phases, in which they encoded and immediately recalled two word lists. In each phase, the critical manipulation occurred during an 8-min delay, where participants either wakefully rested or performed the d2 test of attention. Following the post-encoding activity participants rated how they felt and whether they rehearsed the words. The order of post-encoding activities and the order of presented word lists were counterbalanced across participants. Seven days later, participants performed a surprise free-recall test for both word lists in Session 2.

**Session 1.** As illustrated in Fig. 1, Session 1 included two word-learning trials, which each participant completed one after the other. Each trial consisted of (a) instructions, (b) a short buffer interval, (c) the presentation of a word list, (d) an immediate recall test of the word list, (e) an 8-min post-encoding activity condition (either wakeful-resting condition or d2 test of attention), and (f) a questionnaire measuring mental activity during the previous post-encoding activity. The order of post-encoding activities as well as the order of presented word lists were counterbalanced across participants.

***Word-learning trials.*** In the beginning of each word-learning trial, the experimenter explained instructions for all the following tasks. Participants were instructed to try to remember as many words as possible, in any order, for a recall test immediately following the word list presentation. They were not told that a second recall test would take place. In order to control activity preceding the learning task, each learning trial started with a 2-min buffer during which participants passively listened to ambient music, followed by the visual presentation of the first of two word lists. *Stimuli.* Word lists were derived from the parallel test forms A and D of the verbal learning and memory test (VLMT^40^). Each word list consisted of 15 highly familiar, mono- and bi-syllabic German nouns that were not semantically or phonetically related within and between word lists, and did not rhyme with each other. One after another, each word was presented in the middle of a computer screen for 500 ms followed by an inter-stimulus-interval of 1500 ms (fixation cross).

*Immediate Recall.* Following the word learning task, a picture of a pencil appeared on the computer screen. Participants were instructed to write down as many words as they could remember, in any order, on a sheet of paper with 15 pre-printed lines. Recall time was limited to 1 min. Participants then put the retrieval list into an envelope provided to the left of their workspace.

*Post-encoding activities.* After the immediate free recall of the word list, participants engaged in an 8-min post-encoding activity, which participants spent either wakefully resting or performing a distraction task.

*Wakeful resting.* During the wakeful resting post-encoding condition, the lights in the laboratory were dimmed, the computer screens turned off and participants were asked to rest quietly in their chair and relax for the next 8 min. They were encouraged to close their eyes, to rest their head on the desk and make themselves comfortable. The experimenter stayed in the room at a separate desk and rested with the participants. At the end of the 8-min, they informed the participants that time was up and turned the lights back on.

*Distraction task*. Participants performed an adapted version of the d2-test of attention^41^. The d2, consists of several rows of the letters ‘d’ and ‘p’ with one to four marks above and/or below the letters. Participants were required to cross out as many d’s with two marks as possible within a time limit of 15 s per row. We adapted the task from the original test by adding a second page with an additional 10 rows of the constantly repeating three rows of letter-mark combinations, to match the 8-min wakeful resting post-encoding condition. We employed this task because it requires consistent focused attention, is dissimilar from the main learning task, and requires very limited memory engagement (thus minimising effects of retrieval competition^3^).

*Post-encoding activity questions.* Following each post-encoding activity, participants completed ratings for how often they rehearsed the previously learned words during the preceding post-encoding activity (not at all, once, more than once, the whole time). They also rated on a scale ranging from 0 to 100% to which degree they felt activated (wide awake, stimulated), relaxed, and stressed at this moment or whether they fell asleep (yes, no). See supplementary tables 2 and 3 for an overview of questionnaire items.

**Session 2.** Session 2 occurred exactly 7 days after Session 1. Upon entering the lab, participants first engaged in a 2-min buffer of passive listening to ambient music, after which a surprise free recall test took place. Participants were instructed to write down as many words as possible from both word lists, in any order they wanted, on a sheet of paper with 30 pre-printed lines. Recall time was limited to 2 min. At the end of the session, participants completed two personality questionnaires.

### Questionnaires

*The Highly Sensitive Person (HSP) Scale.* The German translation of the HSP Scale, the HSPS-G^42^ is a translated and adapted version of the HSP Scale^16^, which is a widely used standard 26-item measure to assess SPS and has been used and validated in many different contexts^17^. It measures positive and negative cognitive and emotional responses to various environmental stimuli including art, music, loud noises, smells and fabrics on a 5-point Likert scale ranging from 1 ("does not apply at all") to 5 ("applies completely"). We calculated an SPS score as a mean score of all HSPS-G items. In the present study, the HSPS-G’s Cronbach’s α was 0.87, which is similar to Cronbach’s alphas of previous studies using the original HSPS (α ~ 0.85). Mean and standard deviation of HSPS-G sum scores in our sample (*M* = 76.4, *SD* = 13.69) were similar to those found in larger, more representative samples, previously assessed with the HSPS-G (e.g. Konrad & Herzberg^42^, *M* = 74.21, *SD* = 16.85).

*The Big Five Inventory-SOEP (BFI-S).* As is common practice in SPS studies, Neuroticism (N) was measured as a control for negative affectivity, which is moderately correlated with SPS and may distort HSP Scale scores^43^. We measured N, with a subscale of the BFI-S^44^, a 16-item measure of the Big Five personality factors. The N subscale is comprised of three Likert scaled items, including one reverse-scored item, ranging from 1 ("does not apply at all") to 6 ("applies completely"). The Cronbach’s α for the N subscale was 0.75.

## Results

### Memory retention without SPS as a covariate

Immediate recall performance did not differ between the wakeful resting (*M* = 9.45, *SD* = 2.45) and the distraction (*M* = 9.81, *SD* = 2.47) post-encoding conditions, *t*(63) = − 1.23, *p* = 0.224. To test whether the number of correctly remembered words at Session 2 differed between conditions over time, we conducted a two-way repeated measures ANOVA with recall time (immediate vs. after 7 days) and post-encoding activity (wakeful resting vs. distraction) as within-subjects’ factors. Results showed a significant main effect of time, *F*(1, 63) = 2444.06, *p* < 0.001, *η*_*p*_^2^ = 0.89, indicating that participants remembered less words after 7 days compared to immediately after learning. There was no significant main effect of post-encoding activity, *F*(1, 63) = 3.75, *p* = 0.389, *η*_*p*_^2^ = 0.01, and no significant time* post-encoding activity interaction, *F*(1, 63) = 3.65, *p* = 0.548, *η*_*p*_^2^ < 0.01, indicating that participants forgot a similar number of words in the wakeful resting and distraction condition over seven days.

### SPS’s effects on memory performance

Immediate recall performance was not correlated with SPS neither in the wakeful resting (*r* = 0.03, *p* = 0.814) nor in the distraction condition (*r* = 0.16, *p* = 0.209). To examine whether individual differences in SPS affected differences in the number of correctly recalled words between post-encoding activities over time, we conducted a repeated measures ANCOVA with recall time (immediate vs. after 7 days) and post-encoding activity (wakeful resting vs. distraction) as within-subjects factors and the mean centred SPS scores as a covariate. In within-subject ANCOVAs the probability of a type 1 error is elevated for effects not involving the covariate^45^. Thus, all effects not involving the covariate should be evaluated based on the standard ANOVA model reported above.

Results revealed that there was neither a significant two-way interaction between time and SPS, *F*(1,62) = 0.24, *p* = 0.625, *η*_*p*_^2^ < 0.01, nor a significant interaction between post-encoding activity and SPS,* F*(1,62) = 0.491, *p* = 0.491, *η*_*p*_^2^ < 0.01. The anticipated three-way interaction between time, post-encoding activity and SPS was significant, *F*(1,62) = 7.69, *p* = 0.007, *η*_*p*_^2^ = 0.11, indicating that participants’ level of SPS moderated how the post-encoding activities affected memory retention over the 7 day interval (the time*condition interaction).

**Figure 2:**
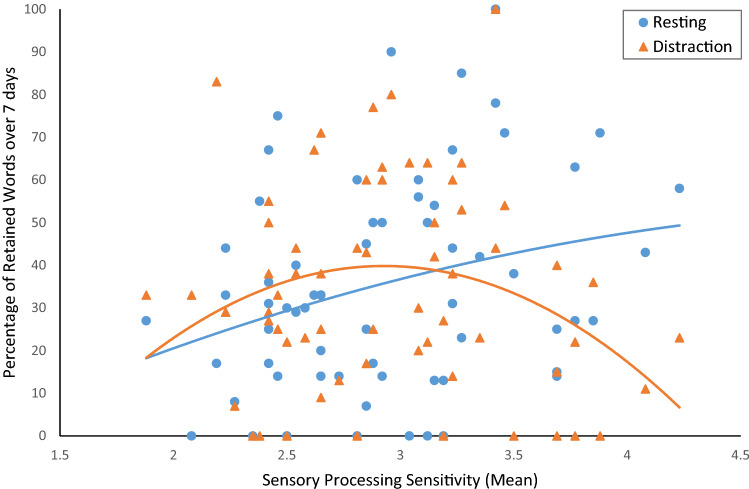
Scatterplots showing the percentage of correctly retained words over the retention interval from immediate to delayed recall after 7 days as a second degree polynomial function of SPS, plotted separately for the wakeful resting (blue circles) and distraction (orange triangles) post-encoding activities.

Next, we plotted the relationship between SPS and participants’ memory retention over time ([delayed recall/immediate recall]*100) separately within each post-encoding condition (Fig. 2). As shown in Fig. 2, the interaction between SPS and memory retention in the distraction condition indicates an unexpected quadratic trend. For the wakeful resting condition, the interaction between SPS and memory retention follows the expected positive linear trend.

To evaluate whether and how SPS predicted memory retention within each of the two post-encoding conditions, we calculated two separate hierarchical regression models for each post-encoding activity. Each model predicts memory retention scores based on participants’ mean-centred SPS scores as a linear term in stage one. Next, a (mean centred) squared SPS-term was entered into the model as a quadratic term in stage two. If participants wakefully rested after learning, the linear SPS-term significantly predicted participants’ memory retention, *F*(1,62) = 5.53, *p* = 0.022, *R*^2^ = 0.08, *β* = 0.29. The addition of the quadratic predictor in stage 2 did not improve the model, indicating that, in the wakeful resting condition, the relationship between SPS and memory retention is linear,* p* = 0.787, *ΔR*^2^ < 0.01.

In the distraction post-encoding condition, the linear SPS-term did not predict memory retention in stage 1, *F*(1,62) = 0.53, *p* = 0.471, *R*^2^ < 0.01, *β* = − 0.09. Adding the quadratic SPS term to the model in stage 2 significantly enhanced the explained variance compared to the linear model, *F*(2,61) = 2.53, *p* = 0.038, *ΔR*^2^ = 0.07. Only the quadratic SPS-term significantly predicted memory retention in the distraction condition, *β* = − 0.27, *p* = 0.038, linear term *p* = 0.953.

In order to check whether the reported analyses and the pattern of results could have been affected by specific influential cases, we repeated the analyses in different subsamples (without participants that retained 0% or 100% memory retention, without participants with more than 60% difference in retention between conditions and others). Under all tested circumstances, the three-way interaction remained significant and the pattern of results persisted. Thus, it is unlikely that the form of the interaction could be the result of potential outlier influences.

As is the case in most studies regarding SPS^17^, there was a significant correlation between SPS and Neuroticism (N), *r* = 0.48, *p* < 0.001. Partialling out N from SPS scores, did not change the positive linear effect of SPS on memory retention in the wakeful resting condition, *F*(1,62) = 5.67, *p* = 0.020, *R*^2^ = 0.08, *β* = 0.29. However, if controlling for N, the quadratic regression model between SPS and memory retention in the distraction condition, no longer reached significance, linear: *F*(1,62) = 0.67, *p* = 0.412, *R*^2^ < 0.01; quadratic: *F*(2,61) = 0.57, *p* = 0.499, *ΔR*^2^ < 0.01.

### Mental activity during the post-encoding activities

Pearson correlations revealed that there was no correlation between participants’ reports on how often they rehearsed parts of the word list and SPS scores, neither in the wakeful resting condition (*r* = 0.07, *p* = 0.592) nor in the distraction condition (*r* = 0.12, *p* = 0.334). Exploratory correlations of the post-encoding activity questionnaire items with SPS scores showed that during the wakeful resting delay, individuals with higher SPS reported feeling less relaxed (*r* =  − 0.31, *p* = 0.012), more stressed (*r* = 0.33, *p* = 0.007) and having more difficulties holding on to their thoughts (r = 0.40, p = 0.001). During the distraction task, participants with higher SPS scores reported that they felt significantly less activated (*r* = − 0.25, *p* = 0.045), more tired (*r* = 0.47, *p* < 0.001), more sleepy (*r* = 0.29, *p* = 0.02) and thought more about solving problems (*r* = 0.34, *p* = 0.006). There were no significant correlations between participants’ answers to any item on the post-condition questionnaire and memory retention scores in any post-encoding activity (*p* > 0.07). See supplementary tables 2 and 3 for in-depth correlation tables for each condition.

## Discussion

In line with expectations, our results demonstrate that individual differences in sensory processing sensitivity (SPS) moderate the interaction between contextual learning conditions and memory retention. If participants were given the opportunity to rest (with their eyes closed while awake) after learning a word list, those with higher (vs. lower) SPS retained more words over a 7-day interval. On the other hand, if participants had to perform a mentally challenging distraction task after learning, SPS showed a curvilinear interaction with memory retention, such that that memory retention for highly sensitive individuals was more hindered, than for their lower SPS counterparts.

This study provides evidence in support of environmental sensitivity theory^25^, as we found that SPS predicts both diminished memory retention under environmental conditions that are generally considered as adverse (distraction after learning), but also greater memory retention under learning-conditions that are generally considered as beneficial (wakeful resting after learning). Although several studies investigated either beneficial or adverse implications of SPS, few studies examined this bipartite nature of the SPS trait within the same subjects.

These individual differences in reactivity to post-learning activity are consistent with fMRI research demonstrating enhanced patterns of resting-state neural connectivity related to depth of processing but also memory-consolidation for individuals with greater levels of sensitivity^27^, as well as a large body of research proposing that depth of processing is a cardinal feature of SPS^17^. As we established previously, this predisposition for deeper more reflective information processing might come at the cost of greater mental effort invested in stimulus processing and post-processing. During quiet, wakeful rest, as sensory processing demands of the environment are reduced to a minimum^46^, spare processing capacity is freed up, allowing higher SPS individuals to engage in increased offline post-processing of previous experiences, thus promoting memory consolidation. Our data supports this line of thought, as higher SPS participants, if they were allowed to rest after learning, not only retained more words over the 7 day interval, but also reported that they felt less relaxed, more stressed and had more difficulty holding on to their thoughts during the wakeful resting delay, indicating greater mental load during the delay.

The critical manipulation differentiating our experimental conditions was the additional attentional processing-demands imposed by the distraction task. We assumed that higher (vs. lower) SPS individuals would invest greater effort in the current distraction task, thus limiting their processing capacity for subconscious memory retention. In line with these assumptions, if participants performed the distraction task after learning, we found that SPS was negatively related to memory retention, and that higher SPS individuals reported thinking more about the task, while also feeling greater fatigue (feeling less activated, more tired and sleepy) during the distraction task.

Interestingly, while we expected this relationship to be linear, as was the case in the wakeful resting condition, in the distraction condition we found a negative curvilinear relationship between SPS and memory retention. Figure 2 displays the three-way interaction between time (immediate, after 7 days), post-encoding activity (wakeful rest, distraction) and SPS affecting memory retention. The form of this interaction indicates that there was only a small difference between post-encoding activities on memory retention for low or medium SPS individuals, but a much larger difference for high SPS individuals. Conspicuously, the point of intersection between regression lines coincides with SPS scores marking roughly 30% of participants with the highest SPS, which are often referred to as “highly sensitive”^23^. Thus, our data suggest that the post-encoding activity had little to no effect on memory retention in the lower-SPS majority of the analysed sample, but much larger effects on memory retention for highly sensitive participants.

The form of this interaction is highly interesting as well, regarding the ongoing debate whether SPS should be considered as a dimensional or categorical trait^17^. While the linear relationship between SPS and memory retention, if participants wakefully rested after learning, supports a dimensional view of SPS; the curvilinear effect of SPS on memory retention in the distraction condition points to a categorical difference between high vs. low SPS individuals. As both premises are true in our sample, our results support the assumption that SPS might be a continuous trait, but roughly 30% of the population falls into a highly sensitive and 70% into a less sensitive group (which could be further divided into a medium and a low sensitive group) along a sensitivity continuum^21,23^.

If we controlled for shared variance between SPS and N (as is common practice in many studies investigating SPS), the negative association between SPS and memory retention in the distraction post-encoding condition vanished, while the positive interaction between SPS and memory retention, in the wakeful resting post-encoding condition, remained robust. This is interesting as within the SPS trait, different aspects of the trait explain different components of the interaction with memory retention. Future theoretical work could use these findings to clarify the relationship between SPS, N and their roles within the environmental sensitivity model. In addition, future work might explore these effects for practical applications with respect to optimal learning environments for individuals with higher SPS, and especially children in the education system.

According to answers to the post-encoding activity questionnaire, higher SPS participants did not actively rehearse words more often than lower SPS participants did. Thus, SPS related higher memory retention following wakeful resting unlikely occurred due to higher active rehearsal of the word lists but rather due to subconscious offline consolidation processes. It is also interesting to note, that higher SPS participants reported lower relaxation and higher stress during the wakeful resting condition. Even though these differences in stress levels do not seem to be related to memory retention, they are well in line with studies showing generally elevated arousal levels for more sensitive individuals^17,47^. Even though our results are clear and consistent with SPS theory and research, further research is needed to replicate and expand on the present findings. For example, our sample consisted of a relatively small sample of highly educated, central Europeans. While the distribution of SPS in our sample was similar to very large and diverse German-speaking samples (e.g.^42,48^), other factors might not have been. As such, generalizability of our findings needs to be confirmed in different samples and populations. We thus recommend a conservative approach to interpretation of the present findings. The nuances of detailed effects reported herein, for example the exact shape of these interactions or a possible inflection point when higher SPS could enhance vulnerability to post-learning distraction should be viewed with caution, until they can be further replicated and confirmed in other samples.

Contrary to some of our own previously-reported findings^49^ and the results of other studies^4^, we were not able to replicate a universal memory benefit of waking rest compared to active wake in the present study. However, as was discussed in the beginning of this article, this was also the case in several other studies conducted in our own and other laboratories^13^. The presented findings might offer a possible explanation for these inconsistencies. Since differences in post-learning activity affect individuals’ memory retention to a greater extent depending on individuals’ SPS levels, the composition of samples regarding participants’ SPS and possibly other individual differences might lead to apparent null effects, if SPS is not controlled for, as was also the case in our sample. Thus, our results support recent calls for combining experimental and correlational approaches to better test and revise theories of long-term memory^1^. This also highlights the importance of digging further into null or contradictory findings to consider whether individual differences could be the cause of differential or unexpected findings.

## Conclusion

Taken together, our results suggest that individual differences in Sensory Processing Sensitivity (SPS) moderate the effectiveness of post-learning activity in regards to memory retention. Higher SPS individuals showed greater memory benefits, if they were allowed to wakefully rest after learning, but they were also more responsive to the negative effects of distracting conditions on memory retention. These findings are in line with environmental sensitivity theory, suggesting that individuals differ in their sensitivity to both adverse as well as supportive (learning) environments.

## Supplementary Information

Supplementary Information.

## Data Availability

The research data of the study are available to the public via the repository Open Science Framework (OSF: https://osf.io/d7csh/?view_only=87d41db4c9f14d439c750247b78ea423).
