# Supplementary material for: Individual differences in sensory processing sensitivity amplify effects of post-learning activity for better and for worse
Source: Sci Rep. 2023 Mar 17;13:4451. doi: 10.1038/s41598-023-31192-9 (PMC10023813; doi:10.1038/s41598-023-31192-9)
Supplement: Supplementary file 1 — Supplementary Information. [file 41598_2023_31192_MOESM1_ESM.pdf]

**Supplementary table 1**. Descriptive statistics and correlations (Pearson) of major study variables.

| | | *M* | *SD* | 1 | 2 | 3 | 4 | 5 | 6 |
|---|---|---|---|---|---|---|---|---|---|
| 1 | Sensory Processing Sensitivity | 2.93 | 0.52 | -- | | | | | |
| 2 | Number of correctly recalled words at immediate recall in the Resting condition | 9.45 | 2.44 | .03 | -- | | | | |
| 3 | Number of correctly recalled words at immediate recall in the Distraction condition | 9.81 | 2.46 | .16 | .55*** | -- | | | |
| 4 | Number of correctly recalled words at the delayed recall in the Resting condition | 3.39 | 2.64 | .27* | .41*** | .25* | -- | | |
| 5 | Number of correctly recalled words at delayed recall in the Distraction condition | 3.51 | 2.73 | .004 | .30* | .53*** | .36** | -- | |
| 6 | Resting condition: Percent of correctly retained words over 7 days | 35 | 24 | .29* | .11 | .10 | .93*** | .25 | -- |
| 7 | Distraction condition: Percent of correctly retained words over 7 days | 34 | 23 | -.09 | .10 | .23 | .28* | .92*** | .22 |

*$p < 0.05$, ** $p < 0.01$, *** $p < 0.001$.

**Supplementary table 2.** Descriptive statistics of post-encoding activity questions about the previous 8-minute *wakeful resting delay* and their correlations (Pearson, Spearman) with SPS and memory retention in the wakeful resting condition.

| | *M* | *SD* | Sensory Processing Sensitivity | Memory Retention (Distraction Condition) |
|---|---|---|---|---|
| in the previous wakeful resting phase I mentally repeated (parts of) the word list | 0,68 | 0,76 | 0,07 | 0,23 |
| I feel activated (wide awake, stimulated) (in%) | 47,36 | 19,08 | 0,00 | -0,02 |
| I feel relaxed (in%) | 66,11 | 21,99 | -0,31* | -0,11 |
| I feel stressed (in%) | 26,55 | 22,76 | 0,33** | 0,17 |
| I had intrusive thoughts | 2,34 | 1,20 | 0,18 | -0,01 |
| I have had rapidly changing thoughts | 2,59 | 1,29 | 0,04 | 0,18 |
| I have had difficulty holding on to my thoughts | 2,20 | 1,18 | 0,40** | 0,16 |
| I have been thinking about others | 3,03 | 1,52 | -0,18 | -0,21 |
| I have been thinking about people I like | 3,13 | 1,53 | 0,03 | -0,07 |
| I have put myself in the shoes of others | 1,97 | 1,13 | -0,08 | -0,12 |
| I have thought about my feelings | 3,19 | 1,55 | 0,11 | 0,00 |
| I have thought about my behavior | 3,08 | 1,30 | -0,10 | 0,03 |
| I have thought about myself | 3,63 | 1,29 | 0,00 | -0,04 |
| I have thought about solving problems | 3,13 | 1,55 | 0,01 | 0,11 |
| I have thought about the future | 3,52 | 1,51 | -0,10 | -0,09 |
| I thought about things I have to do | 3,50 | 1,49 | -0,19 | -0,09 |
| I felt tired | 3,13 | 1,27 | 0,23 | 0,03 |
| I felt sleepy | 3,13 | 1,18 | -0,04 | 0,08 |
| I felt relaxed | 3,67 | 1,07 | -0,39** | -0,11 |
| I felt comfortable | 3,11 | 1,25 | -0,13 | 0,04 |
| I was aware of my body | 3,39 | 1,18 | 0,13 | -0,01 |
| I was thinking about my heartbeat | 1,50 | 0,96 | 0,10 | -0,14 |
| I was thinking about my breathing | 2,30 | 1,48 | -0,14 | -0,10 |
| I felt sick | 1,33 | 0,80 | 0,17 | -0,10 |
| I felt some pain | 1,53 | 1,01 | 0,00 | -0,06 |

* $p < 0.05$, ** $p < 0.01$, *** $p < 0.001$.

**Supplementary table 3.** Descriptive statistics of post-encoding activity questions about the previous 8-minute *distraction delay* and their correlations (Pearson, Spearman) with SPS and memory retention in the distraction condition.

| | *M* | *SD* | Sensory Processing Sensitivity | Memory Retention (Distraction Condition) |
|---|---|---|---|---|
| in the previous d2 phase I mentally repeated (parts of) the word list | 0,44 | 0,75 | 0,12 | -0,14 |
| I feel activated (wide awake, stimulated) (in%) | 59,95 | 19,22 | -0,25* | 0,11 |
| I feel relaxed (in%) | 51,55 | 25,21 | 0,10 | -0,18 |
| I feel stressed (in%) | 36,62 | 24,61 | 0,02 | 0,09 |
| I had intrusive thoughts | 2,03 | 1,18 | -0,12 | 0,07 |
| I have had rapidly changing thoughts | 2,19 | 1,33 | -0,08 | 0,10 |
| I have had difficulty holding on to my thoughts | 2,42 | 1,18 | 0,24 | 0,10 |
| I have been thinking about others | 2,20 | 1,41 | 0,08 | -0,01 |
| I have been thinking about people I like | 2,06 | 1,45 | 0,03 | -0,02 |
| I have put myself in the shoes of others | 1,86 | 1,19 | 0,03 | -0,08 |
| I have thought about my feelings | 2,14 | 1,20 | 0,07 | -0,14 |
| I have thought about my behavior | 2,49 | 1,46 | -0,13 | -0,10 |
| I have thought about myself | 2,89 | 1,42 | 0,09 | -0,02 |
| I have thought about solving problems | 2,86 | 1,48 | ,34** | -0,13 |
| I have thought about the future | 2,06 | 1,41 | -0,03 | -0,02 |
| I thought about things I have to do | 2,17 | 1,44 | -0,09 | 0,03 |
| I felt tired | 2,67 | 1,26 | 0,47*** | -0,06 |
| I felt sleepy | 2,47 | 1,25 | 0,29* | 0,12 |
| I felt relaxed | 2,69 | 1,11 | 0,05 | -0,22 |
| I felt comfortable | 2,58 | 0,96 | -0,01 | -0,13 |
| I was aware of my body | 2,91 | 1,22 | 0,16 | -0,04 |
| I was thinking about my heartbeat | 1,31 | 0,71 | 0,18 | 0,19 |
| I was thinking about my breathing | 1,63 | 1,08 | -0,02 | -0,12 |
| I felt sick | 1,33 | 0,67 | 0,02 | -0,01 |
| I felt some pain | 1,59 | 1,04 | -0,09 | 0,11 |

* $p < 0.05$, ** $p < 0.01$, *** $p < 0.001$.
